# A new point-of-care test for the rapid detection of urinary tract infections

**DOI:** 10.1007/s10096-019-03728-3

**Published:** 2019-11-09

**Authors:** Alyexandra Arienzo, Valentina Cellitti, Valeria Ferrante, Francesca Losito, Ottavia Stalio, Lorenza Murgia, Rossella Marino, Flavia Cristofano, Michela Orrù, Paolo Visca, Salvatore Di Somma, Lorena Silvestri, Vincenzo Ziparo, Giovanni Antonini

**Affiliations:** 1 Science Department, Università degli Studi Roma Tre, Rome, Italy; 2 Interuniversity Consortium “Istituto Nazionale Biostrutture e Biosistemi” (INBB), Rome, Italy; 3 Emergency Medicine, Department of Medical-Surgery Sciences and Translational Medicine, Università La Sapienza of Rome, Azienda Ospedaliera Sant’Andrea, Rome, Italy; 4 Istituto Dermopatico dell’Immacolata, Rome, Italy

**Keywords:** Diagnosis, Infection, POCT, UTI

## Abstract

Urinary tract infections (UTIs) are among the most common infections in all age groups. Fast and accurate diagnosis is essential to ensure a timely and effective therapy. Alongside with reference culture-based methods, several point-of-care tests (POCTs) for early detection of UTIs have been developed, but they have not been significantly implemented in current clinical practice. The Micro Biological Survey (MBS) POCT is a simple test developed by MBS Diagnostics Ltd. (London, UK) for the detection and management of UTIs. The present study has been undertaken to investigate the potentials and limits of the MBS POCT. A total of 349 patients were enrolled in two open-label, monocentric, non-interventional clinical trials in collaboration with an Emergency Medicine department and the outpatient clinic of two hospitals in Rome. Results of urine analysis using the MBS POCT were compared with those of the routine culture-based tests for UTI diagnosis performed by the hospital laboratory. The MBS POCT provided fast results revealing high bacterial count UTIs (≥ 10^5^ CFU/ml) with 97% accuracy, 92% sensitivity, 100% specificity, 99% PPV, and 96% NPV within a 5-h analytical time threshold.

**Electronic supplementary material:**

The online version of this article (10.1007/s10096-019-03728-3) contains supplementary material, which is available to authorized users.

## Introduction

Urinary tract infections (UTIs) remain a major public health problem being among the most common infections in all age groups. The global burden of UTIs is rising, with 16.1% increase in age-standardized incidence between 1990 and 2013 [1, 2]. UTIs are also the most common type of healthcare-associated infection, among which 75% are associated with a urinary catheter, and approximately 20% are cause of bacteremic complications [3, 4].

The current standard for UTI diagnosis is urine culture, followed by antibiotic susceptibility testing (AST) of a midstream, clean-catch urine specimen. UTI patients are empirically treated with antibiotics, and guidelines recommend starting antibiotic treatment before urine culture and AST results become available, delaying by ca. 48 h the initiation of targeted antibacterial therapy [5–7]. Due to the continually changing rates of antimicrobial resistance, empiric treatments do not ensure appropriate stewardship and can result in therapeutic failure [7–9]. Therefore, the empirical antimicrobial regimen of choice should be based on local resistance patterns, as highlighted in various studies from different countries, to effectively prevent the emergence of multi-drug-resistant uropathogens [10–13]. For these reasons, fast and accurate diagnosis, leading to a rational treatment, is essential to achieve a timely and effective therapy.

A point-of-care test (POCT) is defined as a diagnostic tool applicable near the site of patient care that has the potential to provide an accurate and rapid detection of UTIs. Several POCTs for UTI have been developed and are currently commercially available to detect the presence of bacteria or their activity in urine samples, including both culture-based and enzymatic assays, also in automated format [14–18].

The Micro Biological Survey (MBS) POCT is a simple test developed by MBS Diagnostics Ltd. (London, UK) for the management of UTIs [19, 20] (Fig. S1 in Supplementary material). It is a culture-based device that allows semi-quantitative assessment of viable bacteria concentration. Different from other culture-based methods, it measures the enzymatic activity associated with bacterial metabolism, allowing results to be obtained in short time (ca. 5 h).

Performance characteristics of the MBS POCT have preliminarily been investigated in a first prospective diagnostic accuracy evaluation study [21]. A comparative outcome analysis between MBS POCT and reference tests (urine culture and urinalysis, i.e., macroscopic and microscopic examination of urine sediment) was performed, showing that the MBS POCT could detect a suspected UTI within 5 h with high accuracy (90.2%), sensitivity (91.2%), and specificity (89.8%).

Even considering such promising results, more information concerning potentials and limitations of the method was needed. To this purpose, two trials have been undertaken with the following aims: (i) broaden the preliminary results obtained in the first study and (ii) investigate the cases of discordance between MBS POCT and urine culture reference method, with the aim of pointing out strengths and weaknesses of the MBS POCT. The trials have been conducted between 2015 and 2017 in two different clinical settings in Rome: the Emergency Department of “Azienda Ospedaliera Sant’Andrea” (AOSA) enrolling patients with a severe clinical picture and the outpatient clinic of “Istituto Dermopatico dell’Immacolata” (IDI) enrolling community patients enquiring urine culture for either routine screening (e.g., pregnant women) or clinical suspicion of UTI. These two trials gave the opportunity to broaden the diversity and number of tested patients compared to the first clinical trial [21], highlighting the good performance of the MBS POCT.

## Materials and methods

### Study design

A total of 349 patients were enrolled in two open-label, monocentric, non-interventional clinical trials in collaboration with the Department of Emergency Medicine at AOSA, Rome, and the outpatient clinic at IDI, Rome; 101 and 248 patients respectively were enrolled in the two studies.

Enrollment criteria differed between the two trials, due to the different characteristics of patient attending the two hospitals. Patients admitted at AOSA were enrolled between November 2015 and July 2016. Criteria for enrollment were age > 18 years, clinical suspicion of UTI including dysuria or acute suprapubic pain and/or costovertebral tenderness or fever and/or cloudy appearance and/or abnormal color of urine, and/or the presence of a catheter left in place for more than 72 h [21]. Outpatients aged > 18 enquiring for urine culture were enrolled at IDI from May to December 2017.

Prior to study participation, each patient was asked to read carefully through the patient information sheet and sign the informed consent. Approval of both studies was obtained on 14 Jan 2013 from the Ethical Committee of AOSA and on 25 May 2017 from the Ethical Committee of IDI, constituted according to DM 12 May 2006 following Good Clinical Practice. The authorization was given based on the declaration that the patients were duly informed and consenting. In both trials, the MBS POCT results did not imply any change in the normal diagnostic and therapeutic procedures.

### Urine collection

Midstream urine samples or catheter specimens were collected at AOSA hospital 2–4 h after the last void and kept at 4 °C for maximum 2 h prior to analysis. Urine samples at IDI hospital were provided by patients following self-sampling of first morning midstream clean-catch urine specimens [22]. After collection, samples were split into three fractions: one was immediately used for bacterial load assessment with the MBS POCT; one was cultured by the hospital laboratory within half an hour; the last one was split into 1 ml aliquots, each transferred into a 2-ml sterile tube and frozen at − 80 °C after supplementation with 15% (vol/vol) glycerol until eventually used for verification analysis.

### Hospital laboratory tests

Urine culture was the reference method used in both hospitals. Urine cultures were performed by the local microbiology laboratory according to Good Laboratory Practice’s guidelines: 0.010 ml of undiluted and 100-fold diluted urine samples were streaked onto blood agar and BD™ CHROMagar™ Orientation Medium (Becton Dickinson GmbH, Heidelberg, DE) plates using a calibrated loop. Colony counts were performed after at least 24-h incubation at 37 °C. After colony counting, positive results were defined by the presence of ≥ 10^5^ colony-forming units (CFU)/ml.

Bacterial identification and antibiogram were performed using the VITEK® MS and VITEK® 2 systems (BioMérieux Italia S.p.a., Florence, Italy) with 64-well cartridges for antibiotic susceptibility testing (AST) according to the CLSI recommendations [23].

## Bacterial load assessment in urine samples using the MBS POCT

The MBS POCT device is a colorimetric test designed to be used at the patient’s bedside. The test is computer-managed and can be battery powered. It provides specific disposable vials for the detection and quantification of bacteria, which contain a non-selective growth medium called Urine Bacterial Quantification (UBQ) required for analysis. The MBS UBQ vials were produced in compliance with requirements set forth in the EU In Vitro Diagnostic Directive. Three independent production batches of UBQ vials were used throughout the first trial, while two more were used in the second trial.

According to the MBS method, each 1-ml urine sample is manually transferred in a UBQ vial using a disposable, graduated, sterile plastic pipette immediately after urine collection. Bacteriuria is automatically detected upon blue to yellow color change of the medium in the reaction vial during time. Criteria for definition of positive and negative results followed results of previous in vitro studies on artificially contaminated urine samples [19], later confirmed by results from the first clinical trial [21], meaning color change within 5.24 h indicated positivity while slower color change or no color change within analytical timeframe (24 h) indicated negativity. Vials were incubated in the MBS Multireader, which automatically detects the time for color change, at 37 °C. Analyses were performed in duplicate.

### Ex-post verification for bacterial load assessment in urine samples using the MBS POCT

In case of discordance between the urine culture (reference method) results obtained by the hospital laboratory and the MBS POCT results, a verification analysis was performed by personnel of the Microbiology Laboratory of the Science Department, Roma Tre University, Rome, within 2 days from sampling to investigate the source of discordance. Analyses were repeated with both the reference method and the MBS method using urine samples aliquots that had been stored at − 80 °C. Freezing of urine samples did not affect significantly their bacterial load, since no significant decrease of bacterial concentration was observed for frozen samples through 7 days. This trend was observed coherently both using the reference method and the MBS method (data not shown).

Verification analysis of results was performed with the reference method plating 0.010 ml of urine onto non-selective media, including blood agar, CHROM agar, and trypticase soy agar. Colony counting was performed after at least 24-h incubation at 37 °C. Positive results were defined by the presence of ≥ 10^5^ CFU/ml. Positive samples were processed for bacterial identification according to morphology, staining, and biochemical properties of the isolates. Verification analysis of results was performed with the MBS method according to the protocol described above.

In case of concordance between the results obtained with both methods, no further analyses were performed and data was used to carry out statistical analysis. In case of discordance, two possible scenarios were considered (i) when the MBS POCT confirmed a positive result and verification culture showed the absence of a significant bacterial load, the MBS POCT results were definitively considered false positive, and (ii) when the MBS POCT confirmed a negative result and culture showed the presence of a significant bacterial load, results were definitively considered false negative.

### Detection of RAA in urine samples

The residual antimicrobial activity (RAA) test was performed for all discordant samples to check for the presence of antibiotics in urine samples. The detection of residual antibacterial activity was performed using the *Bacillus subtilis* agar disc-diffusion test [24]. An overnight culture of *Bacillus subtilis* ATCC 6051 was plated on a Mueller-Hinton agar dish. A 13-mm sterile filter paper disk was soaked into the urine sample using sterile forceps and, after the excess liquid was eliminated, the disk was poured on the seeded dish. A positive control (disk containing 10 μg ampicillin) and a negative control (blank disk soaked with sterile saline) were also placed on the agar surface. The plates were incubated at 37 °C for 24 h. The presence of a inhibition halo surrounding the disk soaked in urine similar to that observed around the control antibiotic disk was suggestive of the presence of antibiotic activity in urine.

### Diagnostic accuracy evaluation of the MBS POCT

After the verification step, the final results of the MBS POCT were compared with results of the reference urine culture test. Performance characteristics were evaluated by the receiver operating characteristic (ROC) analysis, using the statistical software MedCalc (Windows version 15.0, MedCalc software, Ostend, Belgium) [25]. This analysis is used to determine the validity of a diagnostic test and to define the optimal cut off limit. The area under the ROC curve (AUC) is a measure of how well a parameter can distinguish between two diagnostic groups, i.e., in this investigation, patients with and without a UTI. An area under the curve equal to 1 is that of a test displaying 100% sensitivity and 100% specificity [26, 27].

## Results

### Patient characteristics

The 101 patients attending AOSA Emergency Medicine Department had the following characteristics: 56 (55%) women, 45 (45%) men; mean age 78 years (range 44–96); 79 (78%) catheterized; 77 (76%) were admitted with an ongoing antibiotic therapy and the most frequently used antibiotics were piperacillin-tazobactam (34%), ciprofloxacin (26%) levofloxacin (21%), and cefotaxime (14%), seldom used in combination with other drugs. The 248 outpatients attending the IDI displayed the following characteristics: 173 (70%) women, 75 (30%) men; mean age 65 years (range 18–93); six (2%) catheterized. Of the patients enrolled for the study, only three (1%) were admitted with an ongoing antibiotic therapy.

### Urine culture results

Urine culture routinely performed by hospital laboratories yielded 124 positive results and 220 negative results. Urine culture was not available in three cases, and thus, data from those patients were not included in the statistical analysis. Polymicrobial infections accounted for 21 cases (8%). Bacterial isolates were *Escherichia coli* (*n* = 20), *Enterococcus faecalis* (*n* = 16), *Pseudomonas aeruginosa* (*n* = 7), *Klebsiella pneumoniae* (*n* = 5), *Proteus mirabilis* (*n* = 5), *Enterococcus faecium* (*n* = 2), *Klebsiella oxytoca* (*n* = 2), *Achromobacter xylosoxidans* (*n* = 1), *Enterobacter cloacae* (*n* = 1), *Morganella morganii* (*n* = 1), *Staphylococcus aureus* (*n* = 1), and *Citrobacter freundii* (*n* = 1). The most common polymicrobic associations involved *E. coli* and *E. faecalis* (*n* = 7)*.*

Culture-positive results were obtained from 17 out of 77 urine samples from patients undergoing antibiotic therapy (22%), and from 107 out of 267 samples from patients untreated with antibiotics (40%).

### MBS POCT results

A positive result (median 2.0275, 95% CI 1.7800 to 2.2388) was obtained for 114 out of 344 samples analyzed (33%). The average time for color change of positive samples was 2.03 h (range 0.66–5.24 h). No color change was observed for 85 urine samples, while for 145 samples, a color change was observed between 5.25 and 26 h, and thus reported as negative. MBS POCT-positive results were obtained from 13 out of 77 urine samples from patients undergoing antibiotic therapy (17%), and from 101 out of 267 urine samples from patients untreated with antibiotics (38%). Two urine samples displayed evident macro hematuria which interfered with MBS Multireader measurements. Therefore, the MBS POCT was considered not applicable and the two samples were discarded.

### Diagnostic accuracy of the MBS POCT

Discordance between the results obtained with the reference culture method and the MBS POCT was observed for 19 urine samples out of 344 analyzed (6%). Ex-post verification was therefore carried out on frozen urine samples. Definitive results for all samples following the verification analyses, with the exclusion of two samples with macrohematuria and three samples lacking urine culture results, are summarized in Table 1. Sensitivity, specificity, and positive and negative predictive values of the MBS POCT were 92%, 100%, 99%, and 96%, respectively.

**Table 1:** Summary of definitive results obtained upon verification by urine culture and MBS POCT analysis (dataset from 344 samples)

	Urine culture (cut-off 10^5^ CFU/ml)			
		Positive	Negative	Total
MBS POCT (cut-off 5.24 h)	Positive	113	1	114
	Negative	10	220	230
	Total	123	221	344^a^

^a^Two samples with macro hematuria and three lacking urine culture were excluded from the analysis

**Fig. 1:**
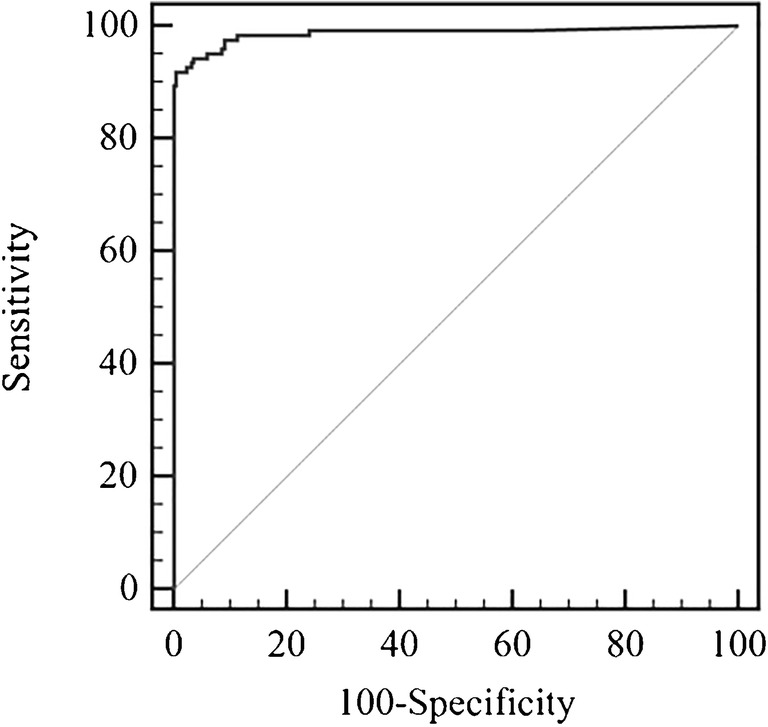
ROC analysis of MBS POCT results (*n* = 344). The ROC curve shows an AUC = 0.987 with 95% confidence interval from 0.973 to 1.000 (dotted line)

**Fig. 2:**
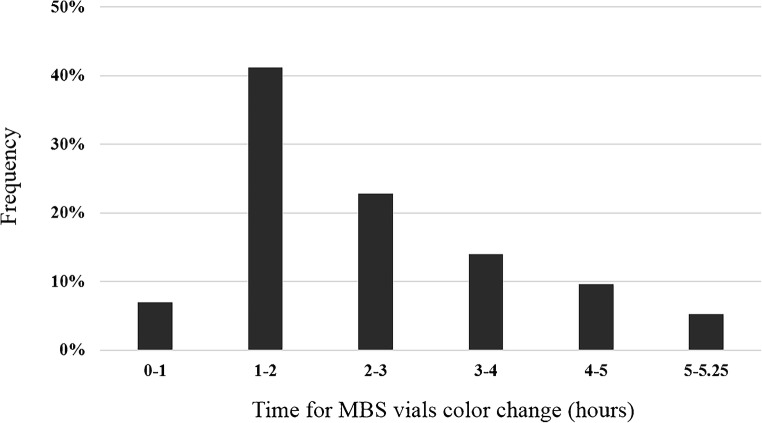
Effect of detection time on MBS POCT positivity. Histograms show detection frequencies (%) of MBS POCT-positive results at hourly intervals

To assess the diagnostic accuracy and optimize the incubation time of the MBS POCT, ROC analysis was performed considering the final results obtained after verification analyses. A total of 344 samples were included in the analysis. Quantitative results, in terms of time taken for the vials to change color, were compared to those of urine culture (either positive or negative). Upon ROC curve analysis, the AUC of the MBS POCT was 0.987 (95% CI 0.973 to 1.000) (Fig. 1), and the associated criterion was 5.24 h. In addition, it should be underlined that almost 50% of culture-confirmed infections were detected within 2 h by the MBS POCT (Fig. 2).

**Fig. 3:**
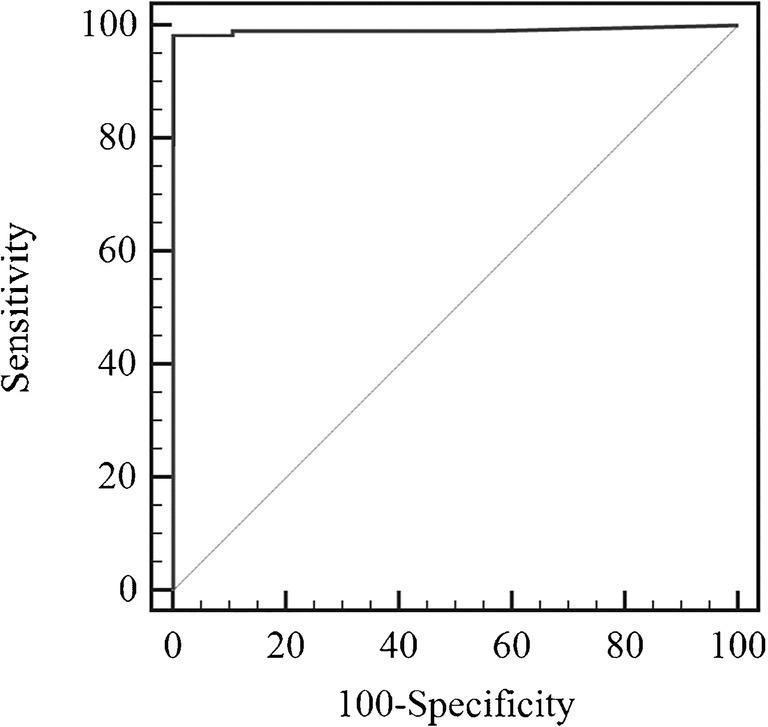
ROC analysis of MBS POCT results (*n* = 303). The ROC curve shows an AUC = 0.992 with 95% confidence interval from 0.979 to 1.000 (dotted line)

**Fig. 4:**
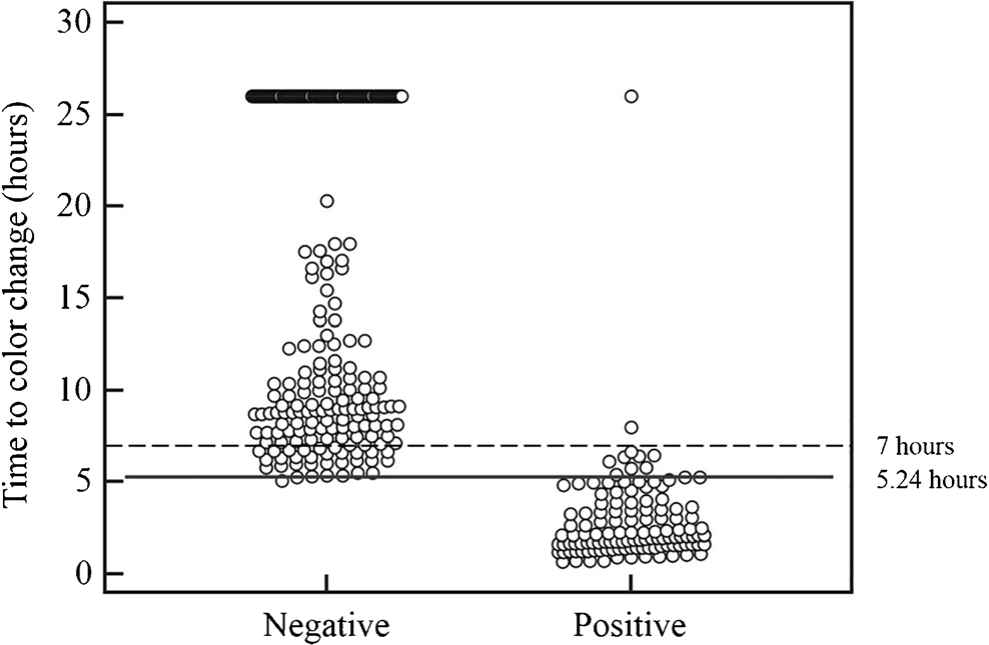
Dot plot analysis of MBS POCT results (*n* = 344). Distribution of positive and negative results with a threshold limit of 5.24 h (sensibility 91.9%, specificity 99.5%). Lines denote the threshold limits, defining three categories: negative (vial color change > 7 h), uncertain (vial color change between 5.25 and 7 h), positive (vial color change ≤ 5.24 h)

Further analysis of discordant cases highlighted an intermediate time zone, ranging from 5.25 to 7 h, which included most of the discordant results. Such intermediate zone is critical, as it comprises samples with borderline bacterial load (around 10^5^ CFU/ml). Therefore, further concordance analysis was performed by combining MBS POCT results for color change times ≤ 5.24 h and ≥ 7 h (Table 2). Sensitivity and specificity considering the combined dataset were 98% and 100%, respectively, whereas positive and negative predictive values reached 100% and 99% respectively. The AUC obtained from the ROC analysis performed on this dataset was 0.992 (95% CI 0.979 to 1.000), and the associated criterion was again 5.24 h (Fig. 3). Categorization of MBS POCT results for cut-off values of 5.24 and 7 h are shown in Fig. 4.

**Table 2:** Summary of definitive results obtained upon verification by urine culture and MBS POCT analysis considering samples showing color change ≤ 5.24 h and ≥ 7 h (dataset from 303 samples)

	Urine culture (cut-off 10^5^ CFU/ml)			
		Positive	Negative	Total
MBS POCT (cut-off 5.24 h)	Positive	113	0	113
	Negative	2	188	190
	Total	115	188	303^a^

^a^Two samples with macro hematuria and three lacking urine culture were excluded from the analysis

## Discussion

In this work, the diagnostic accuracy of the MBS POCT was investigated in different clinical settings in order to highlight the potential benefits and limits of the new method in comparison with conventional urine culture, considered as the gold standard. Different patient cohorts were investigated, i.e., a population of elderly patients admitted to the hospital with underlying disease, often catheterized or undergoing antibiotic therapy (AOSA hospital), together with a cohort of outpatients enquiring for urine microbiological analysis, hence younger, non-catheterized, and without ongoing antibiotic treatment (IDI hospital). This offered the opportunity to define the MBS POCT performance in different clinical frameworks.

Results reported in this study reveal an overall high accuracy, sensitivity, and specificity of the MBS POCT. ROC assessment of the MBS POCT revealed 97% accuracy, and the associated criterion was 5.24 h, coherent with a preliminary study [21]. Moreover, faster response (2 h) was observed for nearly 50% of culture-confirmed UTIs, coherent with the basic principle of the method that implies a reverse correlation between the time of response and the bacterial load in the sample [28, 29]. UTI is the main cause of sepsis in nearly 30% of all septic patients [30], particularly in the elderly population [31]. This type of patients predominated in the Emergency Medicine Department of AOSA; this clinical setting would certainly benefit from a diagnostic tool for early UTI diagnosis, possibly flanking the very first intervention in the “golden hour.”

By comparison with the “golden standard,” MBS POCT true positive and true negative results were mostly comprised within two categories: color change occurring within 5.24 h and after 7 h, respectively. This was confirmed by ROC analysis, which showed very high sensitivity and specificity values (98% and 100% respectively), and 99% accuracy for the above categories. Urines from 41 patients out of 344 (12%) did not fall within these categories, showing vial color change between 5.25 and 7 h. These patients showed a borderline bacterial load, as inferred from viable counts between 10^4^ and 10^5^ CFU/ml in reference urine culture (Table S1 in Supplementary material); setting clear thresholds in these cases is difficult, so that MBS POCT results are uncertain. Irrespective of the analytical method, UTI diagnosis in such patients must be supported by objective examination, taking into account patient symptoms and clinical picture.

MBS POCT results could be of guidance for patient management. For instance, antibiogram and treatment should be secured for patients falling in the ≤ 5.24 h category, as opposed to patients falling in the > 7 h category in which UTI could be ruled out. Of note, monitoring of vials which do not change color within 5.24 h should not be discontinued until 7 h, in order to detect slow-growing bacteria and virtually low bacterial load (< 10^5^ CFU/ml), as this could be significant for some categories of patients. Indeed, the bacterial concentration threshold should be set taking into account patient’s age, sex, and clinical picture [32]. As important as positive predictive value, MBS POCT showed a high negative predictive value (96%), being able to rule out culture-negative patients. As a whole, results obtained in this study have highlighted the robustness of the MBS POCT for the detection of suspected UTIs and suggest how its rapidity, simplicity, and user-friendliness could represent key advantages for the clinical management of patients before a UTI is confirmed by culture-based laboratory methods. By comparison with available devices for UTI detection [33–37], it appears that the MBS POCT could place well among other culture-based devices thanks to its ability to combine short analytical time and high accuracy.

This work also highlights few limitations of the MBS POCT, which appear inherent with the analytical principles of the method. First, since bacterial growth is detected through a colorimetric assay, in the presence of a heavy hematuria, vial color change is biased by the color of the sample, impairing signal detection. This condition is, however, rarely observed in uncomplicated UTIs and was found in our trials in only two cases (0.6%), due to the particular population enrolled in the AOSA study. Furthermore, since the method measures the metabolic activity of bacteria, ongoing antibiotic therapy, hence the presence of antibiotics in urine, affects the viability and/or the metabolic state of bacteria in the sample. This particular condition has been observed in only one case (0.3%). In this case, antibiotic therapy was administered shortly before urine sampling and the infecting bacteria were susceptible. It should be taken into account that urine samples are 10-fold diluted in the MBS POCT, and this can cause significant antibiotic carryover, as opposed to agar plate counting. This situation could have some intriguing clinical implications: when information on ongoing antibiotic therapy is available for a patient, a negative MBS POCT result is suggestive of no infection in urine or successful therapy, while a positive result should warn the clinician about the possible failure of ongoing therapy.

On a different note, setting thresholds for significant UTI is challenging, and the clinical value of a unique 10^5^ CFU/ml cutoff concentration has been questioned. A major concern is the underestimation of infections that could arise from this approach, since low urinary bacterial counts may hold clinical significance in particularly vulnerable categories of patients [38]. Low bacterial counts (10^2^ CFU/ml) can be significant depending on the bacterial agent and patient clinical picture [39], but these infections are usually untreated, though they can degenerate into high-count UTIs in a few days [40]. On this basis, the MBS POCT has some limitations, in that the detection of low-count bacteriuria (< 10^4^ CFU/ml) is not compatible with the rapidity of the test (i.e., detection time < 7 h). Indeed, low bacterial counts can be detected with the MBS POCT device within 15 h (Fig. S2 in Supplementary material), though this does not represent an advantage over routine culture. Another limitation of the MBS POCT is the inability to identify the pathogen(s) involved in the infection, and this could lead to antibiotic misuse. Nevertheless, the MBS POCT could be considered as an enrichment culture that could undergo further analysis according to resource availability, e.g., by direct MALDI-TOF analysis for monomicrobic samples or routine plating for polymicrobic associations, as inferred by microscopy examination of Gram stains of MBS POCT-positive samples [41].

In conclusion, the MBS POCT is a simple and efficient diagnostic tool, which holds promise for improving UTI detection at the patient bedside. Notably, it provides a precise negative predictive value in few hours, allowing early exclusion of high bacterial load UTI diagnosis, with a positive impact on patient management, laboratory workload, and healthcare-associated costs.

## Electronic supplementary material

ESM 1 (PDF 69 kb)
