# Supplementary material for: A new point-of-care test for the rapid detection of urinary tract infections
Source: Eur J Clin Microbiol Infect Dis. 2019 Nov 9;39(2):325–32. doi: 10.1007/s10096-019-03728-3 (PMC7010689; doi:10.1007/s10096-019-03728-3)
Supplement: Supplementary file 1 — (PDF 69 kb) [file 10096_2019_3728_MOESM1_ESM.pdf]

Title: A new point-of-care test for the rapid detection of urinary tract infections.

Journal name: European Journal of Clinical Microbiology and Infectious Diseases.

Authors: Alyexandra Arienzo[1], Valentina Cellitti[2], Valeria Ferrante[2], Francesca Losito[2], Ottavia Stalio[1], Lorenza Murgia[1], Rossella Marino[3], Flavia Cristofano[3], Michela Orrù[2], Paolo Visca[1], Salvatore Di Somma[3], Lorena Silvestri[4], Vincenzo Ziparo[4], Giovanni Antonini[1, 2] #

Affiliations: [1]Science Department, Università degli Studi Roma Tre, Rome, Italy; [2]Interuniversity Consortium "Istituto Nazionale Biostrutture e Biosistemi" (INBB), Rome, Italy; [3]Emergency Medicine, Department of Medical-Surgery Sciences and Translational Medicine, Università La Sapienza of Rome, Azienda Ospedaliera Sant'Andrea, Rome, Italy; [4]Istituto Dermopatico dell'Immacolata, Rome, Italy

Corresponding author:

Prof. Giovanni Antonini, MD PhD, Science Department, Università degli Studi Roma Tre, Viale G. Marconi 446, 00146 Rome, Italy;

Telephone number: 0039 06 5733 6428

Email address: giovanni.antonini@uniroma3.it

Table for Reviewer perusal

Results obtained with the MBS POCT for artificially contaminated urine samples

	MBS time for color change (h)	Standard deviation	Bacterial concentration (Log CFU/ml)
E. coli ATCC 25992	0.64	0.27	9.59
	1.38	0.04	8.59
	3.02	0.08	7.59
	4.42	0.04	6.59
	5.83	0.08	5.59
	6.46	0.24	4.59
	7.97	0.18	3.59
	9.03	0.21	2.65
	9.84	0.02	1.50
	11.09	0.23	0.60
E. faecalis ATCC 29212	0.53	0.08	8.68
	1.44	0.04	7.68
	3.50	0.28	6.68
	4.10	0.00	5.68
	5.77	0.24	4.68
	7.50	0.08	3.68
	8.82	0.16	2.82
	10.59	0.13	1.74
	11.65	0.13	0.78
P. aeruginosa ATCC 27853	0.55	0.07	9.34
	2.27	0.08	8.60
	4.62	0.17	7.86
	5.95	0.16	7.12
	7.07	0.12	6.38
	8.55	0.26	5.64
	9.73	0.13	4.90
	10.38	0.14	4.16
	12.60	0.21	3.42
	13.66	0.08	2.68
S. aureus ATCC 12600	0.38	0.05	8.92
	1.58	0.00	8.18
	2.36	0.21	7.44
	3.30	0.08	6.70
	4.83	0.21	5.96
	6.81	0.08	5.22
	8.85	0.04	4.48
	10.46	0.04	3.74
	12.77	0.04	3.00
	14.50	0.52	2.33
